# Design and Analysis of Fluorine-Free Mold Fluxes for Continuous Casting of Peritectic Steels

**DOI:** 10.3390/ma17235947

**Published:** 2024-12-04

**Authors:** Márcia Maria da Silva Monteiro Pereira, Hervé Tavernier, Tiago dos Santos Junior, Fernando Vernilli

**Affiliations:** 1 Worldwide Materials and Robotics Technologies Centre, Vesuvius Group SA, London EC4A 2AE, UK; herve.tavernier@vesuvius.com (H.T.); tiago.dos.santos@vesuvius.com (T.d.S.J.); 2 Lorena School of Engineering, University of São Paulo, São Paulo 05508-220, Brazil; fernando.vernilli@usp.br

**Keywords:** mold fluxes, peritectic steel, crystallization, viscosity

## Abstract

Fluorine-based mold fluxes are critical for continuous casting of peritectic steels, controlling heat transfer and preventing cracks. However, environmental and health concerns associated with fluorine have spurred the search for alternative flux compositions. This study applied a factorial design to explore the effects of ${\mathrm{Na}}_{2}$O, ${\mathrm{TiO}}_{2}$, $B_{2}O_{3}$, and fluorine on key properties such as viscosity, crystallization temperature, and melting behavior. Analytical methods, including viscosity measurements, differential scanning calorimetry (DSC), X-ray diffraction (XRD), and scanning electron microscopy (SEM-EDS), combined with thermodynamic modeling, were used to evaluate performance. Four formulations were selected based on factorial design results. Sample A, with high ${\mathrm{Na}}_{2}$O, exhibited intense crystallization of merwinite (${\mathrm{Ca}}_{3}{\mathrm{MgSi}}_{2}O_{8}$) and perovskite (${\mathrm{CaTiO}}_{3}$). Sample B, incorporating $B_{2}O_{3}$, had reduced crystallization and suitable viscosity (2.97 Pa·s). Sample C, with a slightly higher fluorine content than Sample B and without $B_{2}O_{3}$, presented balanced low viscosity (1.75 Pa·s) with a moderate crystallization tendency. Sample D, free of fluorine and $B_{2}O_{3}$, showed high viscosity (4.58 Pa·s) and significant crystallization. These results demonstrate that fluorine-free fluxes with properties comparable to fluorine-based compositions can be developed, offering a sustainable alternative for steelmaking. Industrial trials are necessary to validate their performance under operational conditions.

## 1. Introduction

Peritectic steels have important technological applications due to their excellent mechanical strength and toughness. They include High-Strength Low-Alloy grades (HSLA) and the most recent Advanced High-Strength Steels (AHSS), widely used in the automotive industry to reduce vehicle mass while maintaining good structural strength. For example, the influence of hot deformation on the suitability of medium-manganese steels for quenching and partitioning processes, as demonstrated by Krbat’a et al. [1], highlights the importance of refining microstructure and optimizing mechanical properties to meet the demanding performance requirements of these applications. Peritectic grades are also applied in sensitive applications, such as nuclear plants, pipes, and the naval industry [2].

Despite their attractive properties, peritectic steels are among the most challenging to cast via the continuous casting route. These steels solidify in two stages: the peritectic reaction (Liquid steel + δ-Fe →γ-Fe) and the peritectic transformation (δ-Fe →γ-Fe). This process causes significant shrinkage, leading to air gap formation between the steel shell and the mold, reducing heat flux and creating uneven hot spots. These thermal inhomogeneities can cause surface defects or, in severe cases, slab breakout, posing risks to productivity and safety in the casting process [2,3].

Mold fluxes, synthetic slags used in continuous casting, are critical in minimizing such defects. They provide lubrication, control heat transfer, prevent reoxidation, and absorb inclusions. Key properties, including melting speed, viscosity, and crystallization temperature, depend on flux composition and must be tailored to steel grade and casting conditions [3,4]. Traditional fluorine-based mold fluxes, derived from ${\mathrm{CaF}}_{2}$, offer benefits such as reducing liquidus temperature, controlling viscosity, and promoting the crystallization of cuspidine (3CaO.${\mathrm{2SiO}}_{2}$.${\mathrm{CaF}}_{2}$), which decreases horizontal heat flux. The crystalline phase increases thermal resistance, mitigating uneven cooling and reducing crack formation in peritectic steels [5,6,7].

However, fluorine poses significant environmental and safety challenges. ${\mathrm{CaF}}_{2}$ reacts with ${\mathrm{SiO}}_{2}$ to form gaseous ${\mathrm{SiF}}_{4}$, which reacts with water to produce HF and $H_{2}$(${\mathrm{SiF}}_{6}$) (Equations (1)–(3)). These emissions can corrode equipment and pose risks to operators and the environment, leading to growing interest in fluorine-free mold fluxes [8]:

(1) $$2{\mathrm{CaF}}_{2}\left(s\right)+{\mathrm{SiO}}_{2}\left(s\right)\to 2\mathrm{CaO}\left(s\right)+{\mathrm{SiF}}_{4}\left(g\right)$$

(2) $${\mathrm{SiF}}_{4}\left(g\right)+2H_{2}O\left(l\right)\to {\mathrm{SiO}}_{2}\left(s\right)+4\mathrm{HF}\left(g\right)$$

(3) $${\mathrm{SiF}}_{4}\left(g\right)+2\mathrm{HF}\left(g\right)\to H_{2}\left({\mathrm{SiF}}_{6}\right)\left(g\right)$$

Recent research has explored fluorine-free alternatives such as $B_{2}O_{3}$, ${\mathrm{Na}}_{2}$O, and ${\mathrm{TiO}}_{2}$, which exhibit promising properties for use in continuous casting. $B_{2}O_{3}$, for instance, significantly reduces the melting temperature and promotes higher wettability in fluor-free slags compared to fluor-bearing slags by forming more polymerized three-dimensional structures and lowering the liquid flux’s surface tension [9,10]. It also initially decreases viscosity due to the predominance of [${\mathrm{BO}}_{3}$] trigonal structures, but at higher concentrations, it favors the formation of [${\mathrm{BO}}_{4}$] tetrahedral units, which increase viscosity by stiffening the glass network [11].

$B_{2}O_{3}$ also plays a role in influencing crystallization behavior. While it can inhibit crystallization, its presence in fluor-free slags has been shown to enhance the interfacial layer thickness and stability when used with advanced high-strength steels (AHSS), improving wettability compared to fluor-bearing slags [9,12]. Furthermore, the balance of ${\mathrm{Na}}_{2}$O and $B_{2}O_{3}$ concentrations directly affects crystallization temperature and interfacial thermal resistance, which are critical for heat transfer control in peritectic steels [9,10]. ${\mathrm{Na}}_{2}$O, for instance, reduces viscosity and crystallization temperature, complementing the effects of $B_{2}O_{3}$ in enhancing slag properties [13,14,15].

The addition of $B_{2}O_{3}$ to slags alters interfacial properties by reducing surface tension and increasing structural polymerization, impacting stability and thermal transfer during casting. However, the binary basicity also plays a crucial role; higher basicity correlates with increased crystallization temperature and reduced heat transfer rates [16,17,18]. ${\mathrm{TiO}}_{2}$ has also been explored as a fluorine substitute, with the formation of ${\mathrm{CaTiO}}_{3}$ (perovskite) increasing the crystallization rate and improving heat flow control. However, ${\mathrm{TiO}}_{2}$ can form high-melting compounds, such as TiN and Ti(C,N), which may lead to lubrication issues, although some industrial tests have not shown such problems [8,15,19,20]. Yeo et al. [11] confirmed that moderate $B_{2}O_{3}$ levels decrease the degree of silicate polymerization but form rigid structures at higher concentrations, affecting both viscosity and thermal efficiency.

This work aimed to study key properties of fluorine-free mold flux compositions based on different proportions of $B_{2}O_{3}$, ${\mathrm{Na}}_{2}$O, and ${\mathrm{TiO}}_{2}$, considering their application in the continuous casting of peritectic steel slabs. For this purpose, the crystallization behavior of mold flux samples was analyzed through viscosity measurements, X-ray diffraction (XRD), scanning electron microscopy equipped with energy-dispersive spectroscopy (SEM-EDS), differential scanning calorimetry (DSC), and computational thermodynamics simulation. The findings provide valuable data on the effects of $B_{2}O_{3}$, ${\mathrm{Na}}_{2}$O, and ${\mathrm{TiO}}_{2}$ on crystallization kinetics, offering essential insights for developing environmentally friendly mold flux compositions for continuous casting.

## 2. Materials and Methods

### 2.1. Factorial Experimental Design

A factorial experimental design [21,22,23], $2^{4}$, was conducted to determine the compositional limits for fluorine-free mold fluxes for peritectic steels. The amount of oxides (glass formers and network modifiers) and fluorine were considered as independent variables $x_{i}$: ${\mathrm{Na}}_{2}$O ($x_{1}$), $B_{2}O_{3}$ ($x_{2}$), ${\mathrm{TiO}}_{2}$ ($x_{3}$), and F ($x_{4}$); conversely, the response variables ($y_{i}$) were: viscosity ($y_{1}$, dPa·s), crystallization temperature ($y_{2}$, °C), melting temperature ($y_{3}$, °C), and the increase in formulation cost relative to the current standard recipe ($y_{4}$, %). The concentration ranges of other compounds in the ${\mathrm{formulations—SiO}}_{2}$ (26–28 wt%), CaO (26–28 wt%), MgO (5–7 wt%), MnO (3–4 wt%), ${\mathrm{Al}}_{2}O_{3}$ (2–4 wt%), C (5 wt%), and impurities—were fixed and chosen to avoid interference with the independent variables, ensuring that the effects observed could be attributed solely to the selected oxides and fluorine.

The recipes were developed using custom-built software, Versand (v. 7z.001), developed by Metallurgica GmbH & Co.KG (Müllheim, Germany). The viscosity for each composition was estimated using a model based on the Guggenheim’s Quasi-chemical approach [24], while crystallization and melting temperatures were estimated using a factor analysis approach [25]. These models were calibrated and validated through in-house laboratory experiments. The software provided estimations of the dependent variables, based on the stoichiometric chemical formula, which were used in the subsequent optimization steps of the experimental recipes.

The levels for the factorial experimental design are presented in Table 1.

A multiple linear regression was performed based on the factorial experimental design results using the ‘LinearModelFit’ function in Wolfram Mathematica software (version 13.0.1.0, Wolfram Research, Champaign, IL, USA). Second-order interactions between independent variables were included in the model. The complete model is given by Equation (4) [21,26]:

(4) $${\hat{y}}_{i}=b_{0}+\sum_{j=1}^{4}b_{j}x_{j}+\sum_{k=1}^{4}\sum_{l=k}^{4}b_{k,l}x_{k}x_{l}$$

where ${\hat{y}}_{i}$ represents the values predicted by the model for the dependent variables $y_{i}$ (where *i* is 1, 2, 3, or 4), $b_{0}$, $b_{j}$, and $b_{k,l}$ are the regression parameters, and $x_{j}$, $x_{k}$, and $x_{l}$ (where *j*, *k*, and *l* are 1, 2, 3, or 4) are the independent variables. The models were evaluated with hypothesis tests at a 95% confidence level, incorporating *p*-values for the regression (Fisher’s test) and 95% confidence intervals for the parameters (Student’s *t*-test) [21].

The formulation limits were established using the models obtained from the regression step, applying desirability functions within the acceptable ranges for independent variables as required by the steel industry: viscosity (${\hat{y}}_{1}$) from 1.5 dPa·s to 4.5 dPa·s; crystallization temperature (${\hat{y}}_{2}$) from 1050 °C to 1310 °C; melting temperature (${\hat{y}}_{3}$) from 1010 °C to 1210 °C; and cost increase of the recipe (${\hat{y}}_{4}$) below 73%.

Equations (5)–(8) present the desirability functions ($d_{i}$) for each dependent variable (${\hat{y}}_{i}$) [26,27,28,29]:

(5) $$d_{1}=\left\{\begin{array}{cc} 0, & {\hat{y}}_{1}<1.5\;\mathrm{or}\;{\hat{y}}_{1}>4.5\\ \frac{{\hat{y}}_{1}-1.5}{1.5}, & 1.5\leq {\hat{y}}_{1}\leq 3.0\\ \frac{4.5-{\hat{y}}_{1}}{1.5}, & 3.0<{\hat{y}}_{1}\leq 4.5 \end{array}\right.$$

(6) $$d_{2}=\left\{\begin{array}{cc} 0, & {\hat{y}}_{2}<1050\;\mathrm{or}\;{\hat{y}}_{2}>1310\\ \frac{{\hat{y}}_{2}-1050}{120}, & 1050\leq {\hat{y}}_{2}\leq 1170\\ \frac{1310-{\hat{y}}_{2}}{140}, & {\hat{y}}_{2}>1170 \end{array}\right.$$

(7) $$d_{3}=\left\{\begin{array}{cc} 0, & {\hat{y}}_{3}<1010\;\mathrm{or}\;{\hat{y}}_{3}>1210\\ \frac{{\hat{y}}_{3}-1010}{100}, & 1010\leq {\hat{y}}_{3}\leq 1110\\ \frac{1210-{\hat{y}}_{3}}{100}, & 1110<{\hat{y}}_{3}\leq 1210 \end{array}\right.$$

(8) $$d_{4}=\left\{\begin{array}{cc} 0, & {\hat{y}}_{4}>73\\ \frac{73-{\hat{y}}_{4}}{73}, & 0\leq {\hat{y}}_{4}\leq 73\\ 1, & {\hat{y}}_{4}<0 \end{array}\right.$$

The overall desirability *D*, as shown in Equation (9), is calculated as the fourth root of the product of the individual desirabilities ($d_{1},d_{2},d_{3},d_{4}$). This formulation was used to determine the compositional limits for fluorine-free mold fluxes for peritectic steels, ensuring that all response variables met the required industry standards [26,27].

(9) $$D={\left(d_{1}\times d_{2}\times d_{3}\times d_{4}\right)}^{\frac{1}{4}}$$

To identify the composition limits for the recipes, two conditions were considered: one for the fluorine-free formulation and another allowing a reduced fluorine content of 3%. This reduction was proposed due to the conservative nature of some steel plants when adopting new mold flux formulations.

Four recipes were then developed based on the global desirability density plot, selecting formulations within the region of desirability that met the acceptable ranges for independent variables as required by the steel industry. These recipes were subsequently compared.

### 2.2. Materials

In this study, the mold fluxes evaluated include a variety of natural and synthetic raw materials, with granulometry below 75 µm. Silicate sources such as wollastonite and aluminum silicate are combined with secondary raw materials. Fluxing agents including fluorite, sodium carbonate, and boron oxide are incorporated to modify the viscosity and melting characteristics. Titanium dioxide is added to enhance the thermal stability and influence the crystallization behavior of the powder. Additional carbonaceous sources are used to control the melting rate.

The raw materials were mixed in a 3D shaker mixer, Turbula (T2C, WAB, Muttenz, Switzerland), with a capacity for 2 L of mixture. The raw materials were mixed for 15 min, and the final mixture was used for viscosity measurements.

Glassy beads were produced by thermal treatment of the mixture of raw materials produced by the route described above. These mixtures were added to a platinum crucible and held in an electric furnace (Carbolite RHF 16/8, Carbolite, Germany) at 1300 °C for 2 h in air. After melting, the samples were quenched in water at room temperature to obtain glassy beads. The glassy samples were used for chemistry, mineralogy, and thermal analysis.

The techniques used to evaluate the mold fluxes are explained in the following sections.

### 2.3. Methods

#### 2.3.1. Viscosity and Crystallization Temperature Measurements

Glassy powders after melting, i.e., when they are cooling down, experience a smooth change in viscosity around the glass transition temperature (T_g_). This means that the viscosity changes gradually without a sudden shift, resulting in more uniform behavior. Crystalline powders, in contrast, experience a sudden and abrupt increase in viscosity when cooling down due to the crystallization of the molten phase. This change is evident in the log(η) versus 1/T curve, where the slope changes significantly. This transition indicates that the material’s structure is shifting from a liquid (or nearly liquid) state to an ordered solid structure [4].

Slag viscosity measurements were carried out between 1100 °C and 1500 °C in a rotational viscometer (model VIS 413, TA Instruments, Hullhorst, Germany), with analysis capacity up to 1700 °C and control of atmosphere. Between 100 and 200 g of powder were placed in an ${\mathrm{Al}}_{2}O_{3}$ crucible and then in an electric furnace (model CWF 13/13, Carbolite, Neuhausen, Germany) under oxidizing atmosphere at 700 °C for decarburization. After cooling, the material was placed in a platinum crucible for melting at 1350 °C for 40 min in air atmosphere. After cooling, between 60 and 100 g of the resulting slag sample were ground, and of these, 28 g were placed in a platinum crucible and taken to the viscometer. A cooling rate of 10 °C was applied between 1500 °C and 1000 °C. The results of viscosity measurements are plotted on a graph of viscosity (Pa·s) vs. temperature (°C) [3].

#### 2.3.2. X-Ray Diffraction (XRD)

XRD analysis is commonly used to identify crystals formed during crystallization events. These data are crucial for developing new fluorine-free mold flux formulations. It is well known that horizontal heat transfer is predominantly influenced by crystallization events occurring during devitrification and melt crystallization [6,7]. As mentioned earlier, the newly formed crystals must be capable of replacing cuspidine, the primary crystal responsible for heat transfer in commercial mold fluxes.

The evaluation of the amorphous nature of the glass after melting was performed by X-ray diffraction (XRD). Ground glassy beads were used. XRD patterns were recorded at room temperature over 5–75° 2θ angular range with a step size of 0.$01^{\circ }$ and acquisition time of 0.5 s per step. The diffractometer used was a D2 Phaser (Bruker, Karlsruhe, Germany) with a Cu Kα source (λ=1.5406 Å). With this procedure, the sequence of crystal precipitation from melt could be determined.

#### 2.3.3. Scanning Electron Microscopy Coupled with Energy Dispersive X-Ray Spectroscopy (SEM-EDS)

Scanning Electron Microscopy (SEM) analysis can provide further insights into the crystals formed. By examining the crystal morphology and its composition, it is possible, in conjunction with kinetic parameters, to assess the types of crystallization mechanisms.

The composition, morphology, and nature of crystallization products were identified by Scanning Electron Microscopy with Energy Dispersed Spectroscopy analysis (SEM-EDS) (Gemini, Zeiss, Oberkochen, Germany).

Surfaces of fracture of the glassy bead were analyzed for their morphology and concentration of chemical elements, reported in maps for element distribution. The energy used to obtain the X-ray maps was 20 keV, with a spatial resolution of 0.1 µm/pixel.

#### 2.3.4. Differential Scanning Calorimetry (DSC)

DSC, XRD, and SEM together are used to get kinetic data for mold fluxes’ crystallization. The relative crystallinity (α) values for crystallization events can be obtained as a function of temperature (T) from DSC curves through Equation (10):

(10) $$\alpha =\frac{\int_{T_{0}}^{T}\left(\frac{dH}{dT}\right)dT}{\int_{T_{0}}^{T_{e}}\left(\frac{dH}{dT}\right)dT}$$

where *T*, $T_{0}$, and $T_{e}$ are the instantaneous, onset, and end crystallization temperatures, respectively, and dH/dT represents the heat flow rate measured by DSC.

Glassy beads were pulverized and subjected to DSC analysis. The runs were performed using a thermal analyzer STA 449 F3 Jupiter (Netzsch-Gerätebau GmbH, Selb, Germany) with argon acting as purge gas at dynamic conditions. Calibration for the apparatus was performed using α${\mathrm{-Al}}_{2}O_{3}$ as the reference material, building a temperature calibration curve and a sensitivity calibration curve with pure substances. After calibration, the glassy samples were heated up to 1400 °C with a heating rate of 20 °C/min, and then cooled at 30 °C/min. Platinum crucibles with platinum lids were used to minimize the loss of volatile substances. A new baseline was generated for each heating rate using an empty platinum crucible.

Multiple runs were conducted to ensure repeatability, with controlled sample preparation and consistent heating and cooling rates. The heating and cooling rates were kept constant to avoid variations in the crystallization and melting temperatures, ensuring reliable and reproducible results.

### 2.4. Computational Thermodynamic Analysis

To support the analysis of the likely phases to form in each system during cooling, thermodynamic simulations were carried out. The isothermal equilibrium phases were calculated using the Equilib module available from FactSage 8.3 (Thermfact Ltd. and GTT Technologies, Montreal, Canada/Aachen, Germany). The databases used were FToxid, FTmisc, and FactPS. The phase stability diagrams were calculated in a temperature range of 1000 °C to 1400 °C. In this study, binary basicity, ${\mathrm{TiO}}_{2}$, ${\mathrm{Na}}_{2}$O, and $B_{2}O_{3}$ were not added individually to a base system but rather combined in specific proportions to create the tested formulations, as detailed in Table 1, with the goal of evaluating their effects on slag activities and crystallization behavior.

## 3. Results and Discussion

The results obtained in the simulation of the formulations for the factorial design with experimental levels in Table 1 are presented in Table 2.

Multiple linear regression was performed on the data from Table 2 according to Equation (4), resulting in Equations (11)–(14). These equations account for a 95% confidence level according to Student’s *t*-test for the exclusion of non-significant parameters and digits:

(11) $${\hat{y}}_{1}=5.7-0.31x_{1}-0.5x_{2}+0.36x_{3}-1.0x_{4}+0.03x_{1}x_{2}-0.02x_{1}x_{3}+0.05x_{1}x_{4}-0.05x_{2}x_{4}$$

(12) $${\hat{y}}_{2}=1112-15x_{1}-6x_{2}+22x_{3}-29x_{4}+1.6x_{1}x_{4}$$

(13) $${\hat{y}}_{3}=1299-20x_{1}-3x_{2}-2x_{3}+5x_{4}-0.5x_{1}x_{4}$$

(14) $${\hat{y}}_{4}=23+5x_{2}+7.4x_{3}-0.7x_{1}x_{2}+0.5x_{3}x_{4}$$

The hypothesis test for regression presented the following results: $R^{2}=0.9983$ and *p*-value = $2.7\times 10^{-13}$ for Equation (11); $R^{2}=0.9868$ and *p*-value = $7.7\cdot 10^{-9}$ for Equation (12); $R^{2}=0.9975$ and *p*-value = $1.9\cdot 10^{-12}$ for Equation (13); and $R^{2}=0.9821$ and *p*-value = $3.4\cdot 10^{-8}$ for Equation (14). The results indicated that all variables and interactions presented in Equations (11)–(14) are significant (*p*-value < 0.05) at a 95% confidence level according to Fisher’s test, and that more than 98% of the variance of the independent variables ($R^{2}>0.98$) can be explained by the independent variables and their interactions in the regression model, indicating a good fit of Equations (11)–(14) to the data.

**Figure 1:**
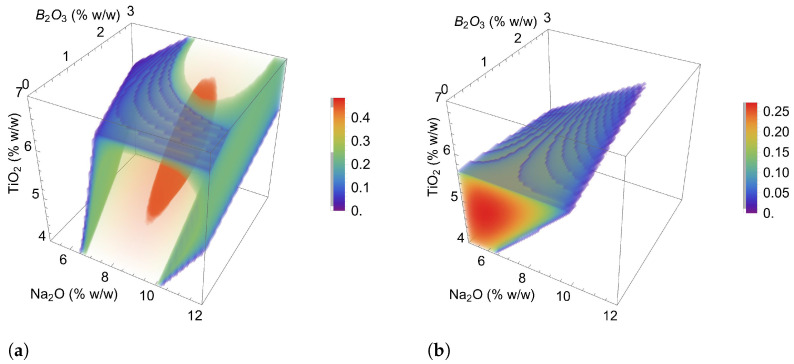
Density plot for the desirability function for formulations containing: (**a**) 0% fluorine and (**b**) 3% fluorine.

Figure 1 presents the desirability graphs as a function of the mass percentages of ${\mathrm{Na}}_{2}$O, ${\mathrm{TiO}}_{2}$, and $B_{2}O_{3}$ for formulations containing (a) 0% fluorine and (b) 3% fluorine. These graphs are used to evaluate the limits within which the formulation composition can be varied and the impact of these changes on desirability.

Figure 1a shows the region (color-coded from green to red) where the highest desirability values can be achieved for formulations with 0% fluorine. These optimal values occur for formulations containing 8–10% ${\mathrm{Na}}_{2}$O, 4–6% ${\mathrm{TiO}}_{2}$, and 0–3% $B_{2}O_{3}$. This result is useful for tailoring formulations to different continuous casting conditions and requirements.

Figure 1b illustrates the region where formulations meet specifications with a fixed 3% fluorine content. It is observed that the maximum desirability values in Figure 1b are lower than those in Figure 1a, indicating a narrower range for meeting all constraints. This is because the 3% fluorine formulations are closer to the standard formulation, reducing the necessary amounts of ${\mathrm{Na}}_{2}$O, ${\mathrm{TiO}}_{2}$, and $B_{2}O_{3}$ to meet continuous casting process conditions.

Based on the results obtained from the search for the region containing the best formulations, four compositions in the high-desirability regions for fluorine-containing and non-fluorine-containing density plots were chosen. The chemical compositions of the samples are presented in Table 3.

According to Mills [30], the factors that influence the mold fluxes’ melting rate are the free carbon content, the type and size of the carbon particle, the carbonate content, and the presence of exothermic constituents. In this study, to avoid variation in the melting speed of the samples, no exothermic materials were used for this study and the free carbon of the samples was kept constant at 5%.

**Figure 2:**
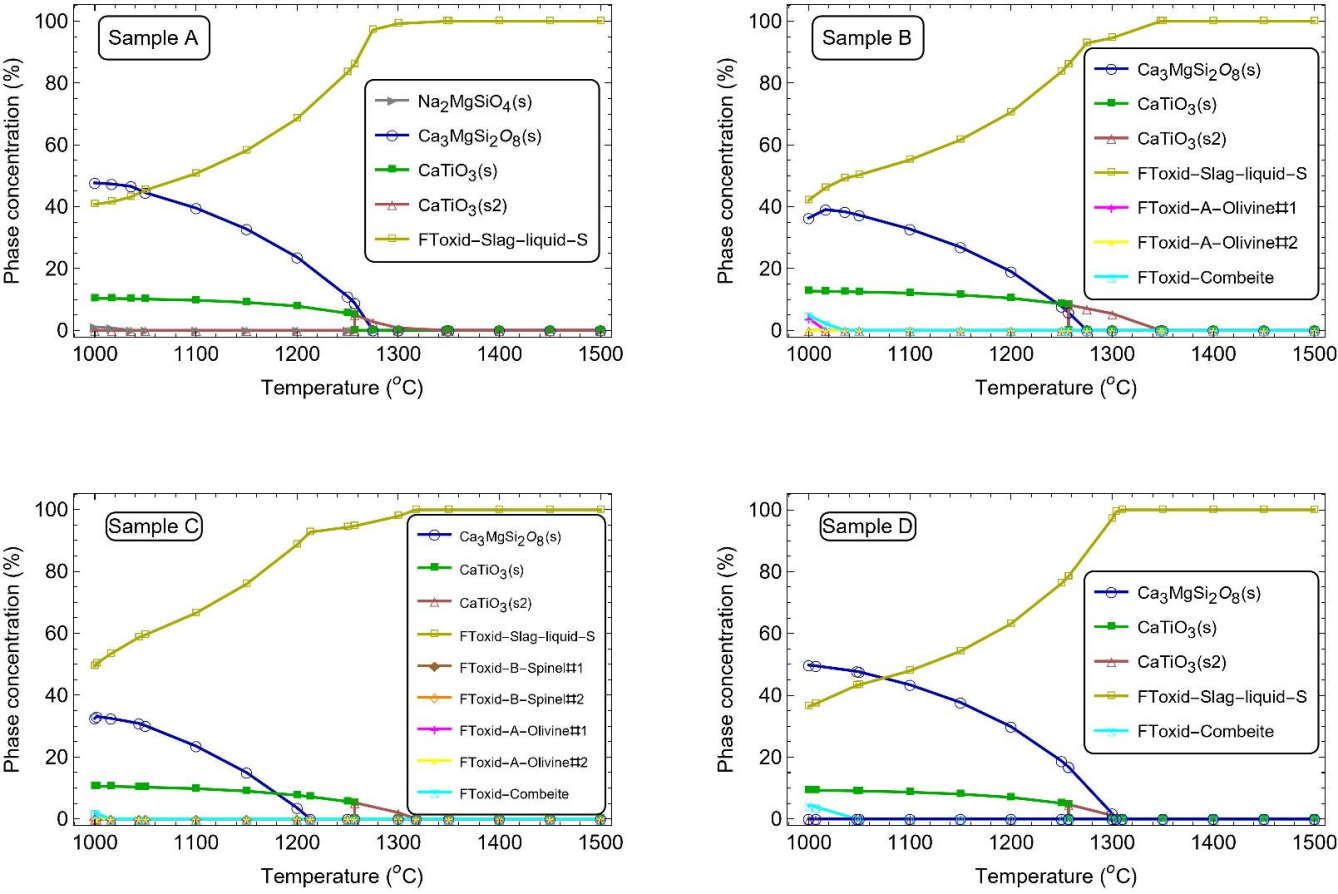
Evolution of phases in equilibrium for the compositions studied in this work, estimated by thermodynamics calculations.

The expected phases in equilibrium between 1000 °C and 1500 °C, estimated by thermodynamics calculations by FactSage 8.3 (Thermfact Ltd. and GTT 279 Technologies, Montreal, Canada/Aachen, Germany), see Figure 2, suggest that all the systems studied in this work would crystallize, with the first crystals being stable below 1300 °C. Samples A and D would achieve the highest crystallization rates at lower temperatures, exceeding 60 wt% of the mass at 1000 °C. The main crystal formed should be merwinite (${\mathrm{Ca}}_{3}{\mathrm{MgSi}}_{2}O_{8}$), followed by perovskite (${\mathrm{CaTiO}}_{3}$).

All samples exhibit stability of ${\mathrm{Ca}}_{3}{\mathrm{MgSi}}_{2}O_{8}$ at lower temperatures, though this stability diminishes at higher temperatures. In samples A, B, and D, ${\mathrm{Ca}}_{3}{\mathrm{MgSi}}_{2}O_{8}$ stabilizes at earlier stages compared to sample C. This can be explained by the higher amount of F in sample C, which increases the quantity of liquid in the system. In samples A, C, and D, ${\mathrm{CaTiO}}_{3}$ is stabilized at a similar temperature, 1300 °C. On the other hand, sample B is stabilized at a higher temperature (1347 °C), which can be explained by the higher ${\mathrm{TiO}}_{2}$ content in its formulation.

From the rheology measurements, the crystallization temperature and viscosity at 1300 °C were obtained and reported in Table 4. The crystallization temperature was defined as the onset of viscosity increase during the cooling down of the molten slags.

When comparing the results for the four compositions, samples without fluorine had a higher crystallization temperature. Despite the earlier crystallization, viscosity values at 1300 °C were not higher for sample A, but it was the highest for sample D. This difference in viscosity highlights the effect of ${\mathrm{Na}}_{2}$O in the reduction of the melt viscosity: for composition A, ${\mathrm{Na}}_{2}$O was higher (9.40 wt%) compared to the quantity of this oxide in composition D (8.0 wt%).

The observed differences in the results can be attributed to variations in oxide compositions, such as $B_{2}O_{3}$ and ${\mathrm{Na}}_{2}$O, which influence viscosity and crystallization temperatures. The binary basicity also plays a significant role in crystallization [31], with higher basicity leading to increased crystallization temperatures. Additionally, ${\mathrm{TiO}}_{2}$ can replace fluorine, affecting both the crystallization rate and heat flux.

According to the literature, $B_{2}O_{3}$ and ${\mathrm{Na}}_{2}$O can substitute fluorine, reducing viscosity and crystallization temperatures [3,14,32]. This is evident in Samples A and B, which have similar viscosities but different crystallization temperatures. Sample B includes $B_{2}O_{3}$, which decreases viscosity and impedes slag crystallization. These effects are unfavorable for the continuous casting of peritectic steels. Sample B also has the highest ${\mathrm{TiO}}_{2}$ content and a small amount of fluorine. The differences in crystallization behavior can be further influenced by the effect of binary basicity, as higher basicity results in higher crystallization temperatures. Sample A, with its high ${\mathrm{Na}}_{2}$O and ${\mathrm{TiO}}_{2}$ content, exhibits a high crystallization temperature.

Sample C shows an intermediate crystallization temperature and lower viscosity compared to the other samples. This is explained by its higher basicity, absence of $B_{2}O_{3}$, and the presence of fluorine.

Sample D has a higher viscosity than Samples A, B, and C, and a crystallization temperature similar to Sample A. This can be attributed to the absence of fluorine and $B_{2}O_{3}$ in its composition.

**Figure 3:**
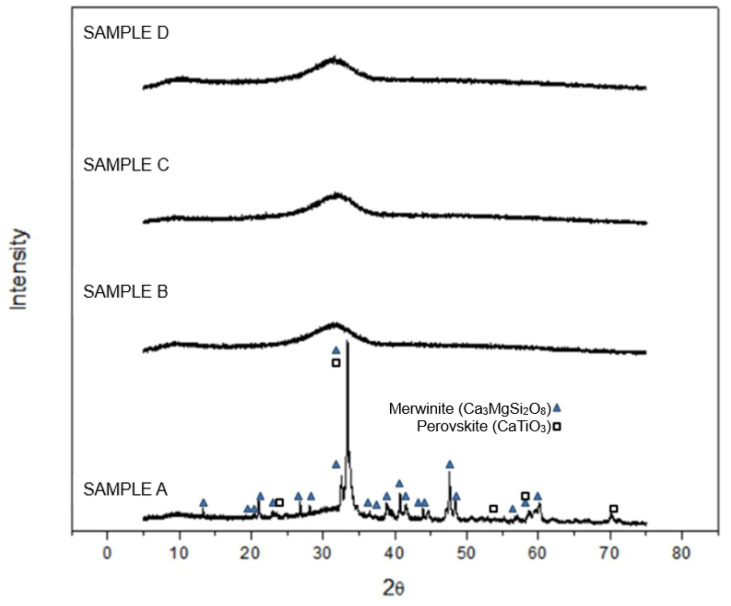
XRD of the powder glass samples of samples A, B, C, and D.

The XRD diffractograms of the powder glassy samples obtained after melting and quenching (Figure 3) indicate that samples B, C, and D were completely amorphous. In contrast, the pattern of sample A, which shows defined diffraction peaks, indicated the formation of merwinite (${\mathrm{Ca}}_{3}{\mathrm{MgSi}}_{2}O_{8}$) and perovskite (${\mathrm{CaTiO}}_{3}$) during cooling, as also forecasted by the thermodynamics simulation. However, the amount of the glassy phase in the sample seemed to be significant, as indicated by the high intensity of the amorphous halo. This result demonstrates that the melt derived from the sample A composition has a higher tendency to crystallize on cooling, even at high cooling rates.

The presence of crystals in sample A is associated with the higher presence of ${\mathrm{Na}}_{2}$O in this composition. In the work of Riaz et al. [33], the effects of ${\mathrm{Na}}_{2}$O on the crystallization behavior and the heat transfer of ${\mathrm{CaO-SiO}}_{2}{\mathrm{-Na}}_{2}{\mathrm{O-B}}_{2}O_{3}{\mathrm{-TiO}}_{2}{\mathrm{-Al}}_{2}O_{3}{\mathrm{-MgO-Li}}_{2}$O slags were investigated. CCT (Continuous Cooling Transformation) and TTT (Time-Temperature-Transformation) diagrams showed that the increase in ${\mathrm{Na}}_{2}$O content increased the critical cooling rate needed to keep a fully amorphous structure of the solid. Thus, the incubation time for nuclei formation is shortened, and the tendency to crystallization is increased. While the thermodynamics calculation pointed out sample D as a system with a higher quantity of stable crystals below 1300 °C, this was not observed in this study. The reason for this discrepancy between thermodynamic equilibrium and the real experiment is the kinetics. Sample D was very viscous, which could hinder the transport of matter from the glassy matrix to the areas where crystal nuclei were formed.

**Figure 4:**
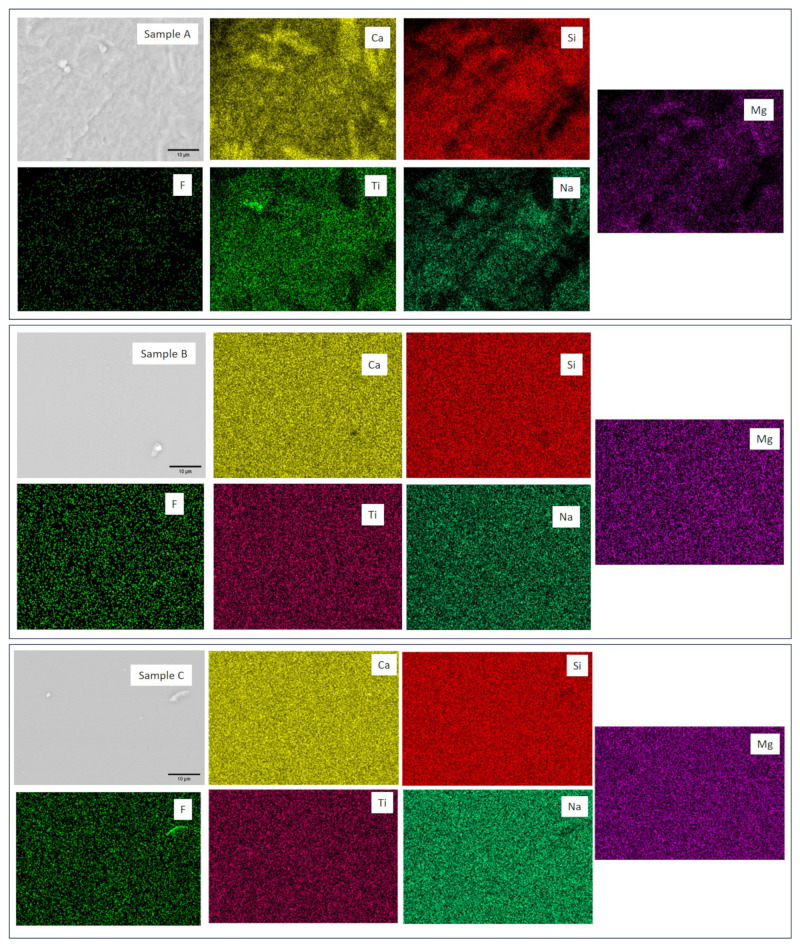

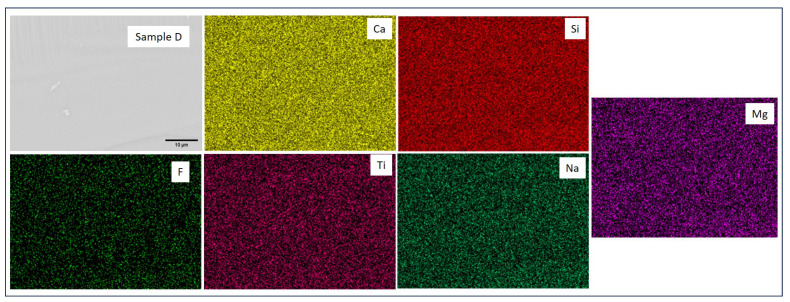
SEM images of the polished sections of samples A, B, C, and D.

The microstructural analysis of the glassy samples, combined with EDS (Figure 4), also revealed the presence of crystals dispersed in the vitreous matrix (Figure 4, Sample A). Elemental mapping of the observation area shows the concentration of Ca, Ti, Si, and Mg in specific regions, illustrating the dispersion of crystalline phases (${\mathrm{CaTiO}}_{3}$ and ${\mathrm{Ca}}_{3}{\mathrm{MgSi}}_{2}O_{8}$) in the vitreous matrix.

This observation is consistent with the XRD analysis, which also identified perovskite as a significant crystalline phase. This alignment between morphological analysis and XRD results strengthens the conclusion regarding the crystallographic composition of Sample A.

On the other hand, no defined crystalline morphologies and a higher concentration of elements in specific regions were observed in samples B, C, and D. This also corroborates the XRD results, indicating that these compositions were amorphous after solidification by quenching in water.

**Figure 5:**
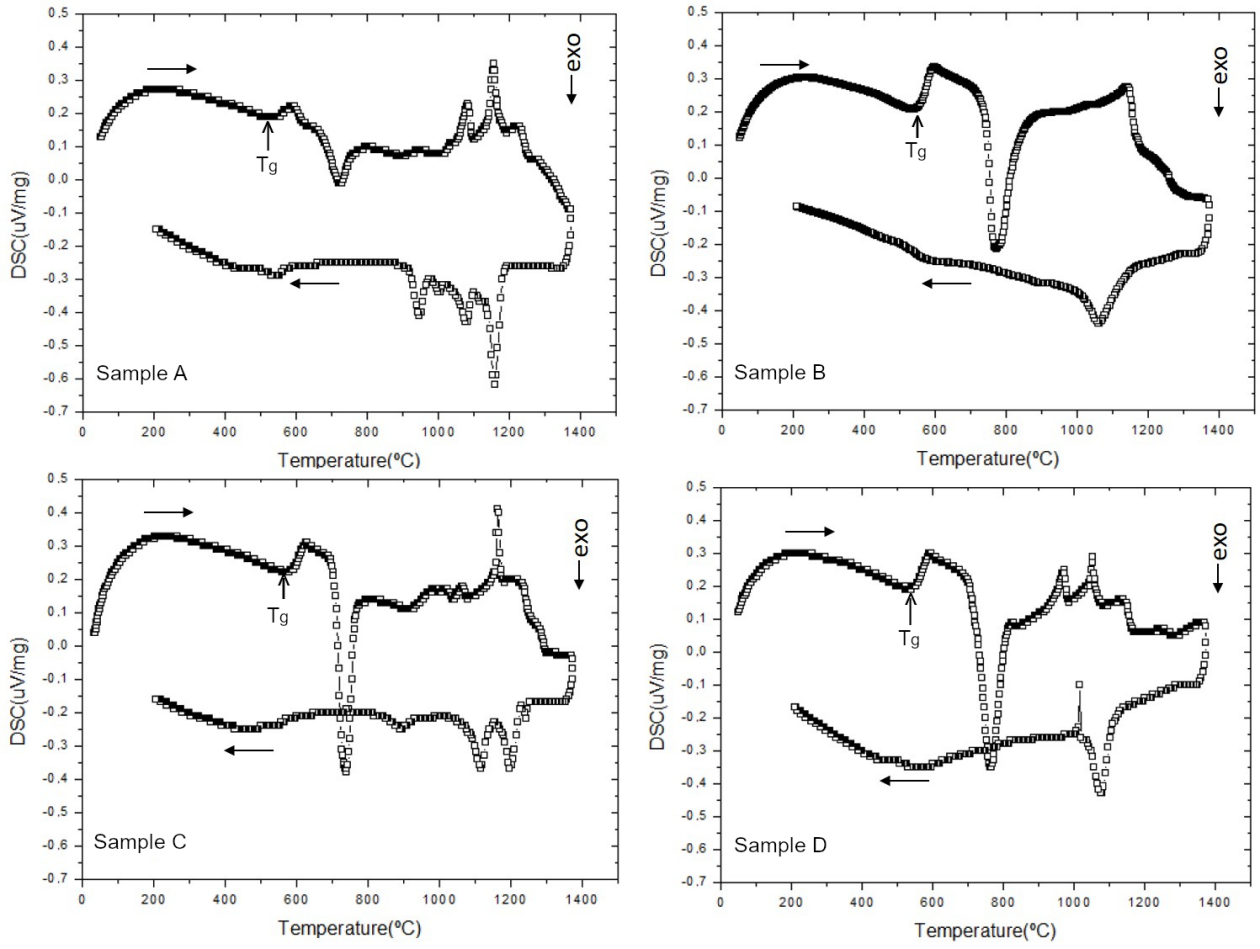
DSC curves of the samples A, B, C, and D, obtained at a heating rate of 20 °${\mathrm{C\cdot min}}^{-1}$ and a cooling rate at 30 °${\mathrm{C\cdot min}}^{-1}$.

As pointed out by the thermodynamics simulation, all four compositions evaluated would present crystals in equilibrium at lower temperatures. To investigate the crystallization likelihood for these systems, DSC measurements were carried out under controlled heating and cooling rates. Figure 5 shows DSC curves for a cooling rate of 30 °C/min for the investigated samples.

The DSC analysis for Samples A, B, C, and D provides insights into the thermal transitions and crystallization behaviors of the samples as the temperature changes.

The presence of endothermic peaks indicates points where the sample absorbs heat, corresponding to glass transitions or melting. Exothermic peaks indicate heat release associated with crystallization or recrystallization of new phases. The temperature and intensity of these peaks provide information on the thermal stability and crystallization behavior of the materials.

For all the compositions, during heating, three main thermal-activated phenomena were observed. Firstly, the increase in heat absorbed by the samples, corresponding to its glass transition temperature ($T_{g}$), followed by the intense release of heat associated with crystallization, and later, several heat-absorption peaks associated with the melting of the samples.

In Table 5, the onset temperatures for glass transition, crystallization, and initial melting during the heating cycle are listed. Despite the $T_{g}$ temperature not differing much between the samples, the crystallization temperature for Sample A was lower than for the other samples. Also, the amount of heat released for this sample, associated with crystallization, was less than for the other systems. From the XRD and SEM results (Figure 3 and Figure 4, respectively), Sample A already presented a significant number of crystals; thus, the amorphous volume of this material was lower compared to Samples B, C, and D. Consequently, the crystallization during heating was lower in Sample A.

The temperature range for melting was similar for the four compositions, although the thermal events associated with this range (endothermic peaks) differed in number and intensity. The maximum temperature reported in the melting range agrees with the one observed in the thermodynamics simulations for these systems in equilibrium, when 100 wt% of liquid is formed (Figure 5).

During the cooling down, significant exothermic events were identified in the DSC curves. For Sample A, the cooling curve shows a distinct downward exothermic peak between 1200 °C and 900 °C, indicating significant crystallization events. For Sample B, the cooling curve has a less pronounced downward exothermic peak in the range between 1200 °C and 1000 °C, suggesting a lower tendency to crystallize compared to the other samples, which can be explained by the presence of $B_{2}O_{3}$. Sample C showed a noticeable and moderately sharp downward exothermic peak (1250–1050 °C) while cooling, indicating a good tendency to crystallize. Finally, the cooling curve for Sample D shows a pronounced downward exothermic peak (1200–1000 °C), indicating a strong tendency to crystallize.

**Figure 6:**
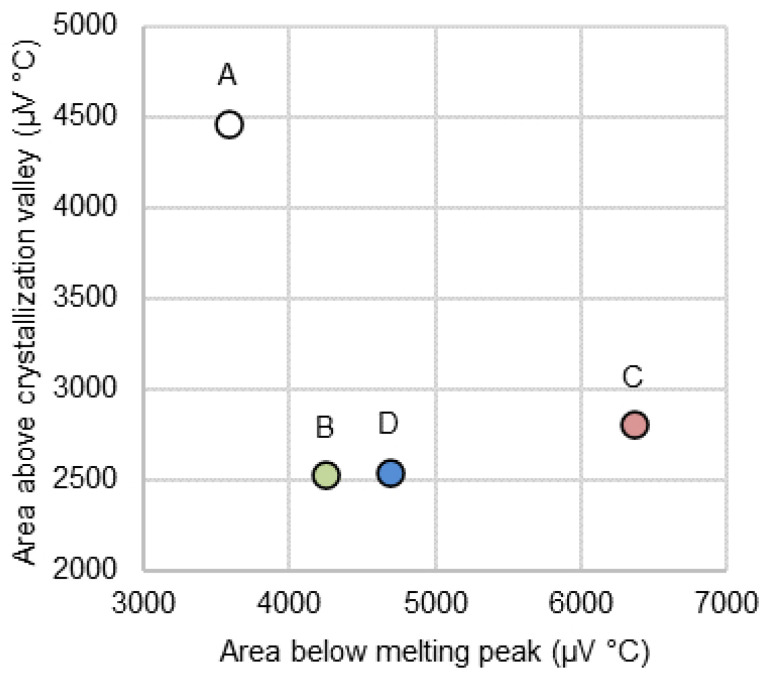
Comparison between melting and crystallization areas measured from DSC curves for samples A, B, C, and D.

A comparison of the areas below and above the endothermic and exothermic peaks, related to melting and crystallization (during cooling down), respectively, can be seen in Figure 6. Given that, from thermodynamics simulations, the main crystals expected to be formed in all the systems are the same (${\mathrm{CaTiO}}_{3}$ and ${\mathrm{Ca}}_{3}{\mathrm{MgSi}}_{2}O_{8}$), the quantity of energy released during crystallization might be associated with the relative degree of crystallinity of each system. In this case, Sample A would be the system with the highest degree of crystallinity compared to the other compositions evaluated. This trend agrees with the XRD and SEM results and shows that a fluorine-free composition with similar rheological behavior and a high degree of crystallinity can be obtained if fluorine is replaced by ${\mathrm{Na}}_{2}$O and ${\mathrm{TiO}}_{2}$.

## 4. Conclusions

This study employed a factorial design to investigate the impact of ${\mathrm{Na}}_{2}$O, ${\mathrm{TiO}}_{2}$, $B_{2}O_{3}$, and fluorine on four dependent variables: viscosity, crystallization temperature, melting temperature, and cost increase. The factorial design, combined with the desirability function, helped define acceptable ranges for these oxides, ensuring that the resulting formulations met industry criteria for these key properties. Based on these acceptable ranges, the four samples A, B, C, and D were formulated and evaluated.

Sample A was designed to be fluorine-free and $B_{2}O_{3}$-free, resulting in a high ${\mathrm{Na}}_{2}$O content and higher basicity. This formulation exhibited a strong tendency for crystallization, as evidenced by the XRD and SEM-EDS analyses, which identified the formation of merwinite (${\mathrm{Ca}}_{3}{\mathrm{MgSi}}_{2}O_{8}$) and perovskite (${\mathrm{CaTiO}}_{3}$). Thermodynamic calculations supported these findings, showing that merwinite was the dominant crystalline phase at lower temperatures. This sample is ideal for applications where significant crystallization is desired.

Sample B was designed to reduce the fluorine content by incorporating $B_{2}O_{3}$ and increasing the ${\mathrm{TiO}}_{2}$ concentration. This resulted in a formulation with reduced crystallization tendency. The DSC analysis showed a less pronounced exothermic peak, indicating lower crystallization. Thermodynamic simulations suggested that merwinite stabilized at lower temperatures, while perovskite formed at higher temperatures (above 1347 °C). Sample B would be beneficial in scenarios where lower crystallization and reduced viscosity are preferred.

Sample C was formulated with a slightly higher fluorine content than Sample B and without $B_{2}O_{3}$. This composition achieved a balanced performance, with lower viscosity and a favorable crystallization temperature. The DSC analysis showed a noticeable exothermic peak, indicating a good crystallization tendency. Thermodynamic results showed that merwinite was the primary crystalline phase at lower temperatures. This sample offers a good balance of properties for industrial applications but did not show crystal formation in quenching experiments, indicating a need for further testing.

Sample D was designed to achieve a high-viscosity flux, without fluorine or $B_{2}O_{3}$. This formulation exhibited the highest viscosity, as expected, and the DSC curve displayed a pronounced exothermic peak, signaling a strong crystallization tendency. Thermodynamic calculations revealed that merwinite and perovskite stabilized at higher temperatures compared to the other samples. This formulation emphasizes the trade-off between high viscosity and substantial crystallization, making it suitable for specific applications requiring both high viscosity and crystallization.

In conclusion, the factorial design and desirability function effectively identified the acceptable ranges for ${\mathrm{Na}}_{2}$O, ${\mathrm{TiO}}_{2}$, $B_{2}O_{3}$, and fluorine, resulting in four distinct mold flux formulations. The best formulation depends on the desired properties for specific industrial applications. Considering the challenges faced while casting peritectic steels, this study has shown that fluorine-free mold flux compositions with similar properties to the traditional fluorine-based ones can be designed. Nevertheless, the transfer of heat through the molten film of a fluorine-free flux also needs to be assessed under industrial conditions to validate this solution as a better alternative to fluorine-based products.

## 5. Further Works

For further work, Continuous Cooling Transformation (CCT) and Time-Temperature-Transformation (TTT) diagrams will be developed to better illustrate crystallization behavior. Additionally, a test in a steel plant is planned to confirm the results.

## Figures and Tables

**Table 1:** Levels for the factorial experimental design to determine the recipe composition range.

Level	${\mathrm{Na}}_{2}$O (%)	$B_{2}O_{3}$ (%)	${\mathrm{TiO}}_{2}$ (%)	F (%)
Lower (−)	5	0	4	0
Higher (+)	12	3	7	3

**Table 2:** Results obtained with the simulation of the mold fluxes’ formulations.

Exp.	$x_{1}$	$x_{2}$	$x_{3}$	$x_{4}$	$y_{1}$	$y_{2}$	$y_{3}$	$y_{4}$
1	−	−	−	−	5.1	1135	1188	54
2	+	−	−	−	2.3	1017	1054	51
3	−	+	−	−	3.9	1111	1181	58
4	+	+	−	−	1.7	1014	1041	43
5	−	−	+	−	5.9	1186	1182	74
6	+	−	+	−	2.4	1083	1046	74
7	−	+	+	−	4.8	1171	1188	79
8	+	+	+	−	2.0	1079	1035	66
9	−	−	−	+	2.9	1064	1197	57
10	+	−	−	+	1.1	987	1049	60
11	−	+	−	+	2.3	1045	1192	62
12	+	+	−	+	0.9	971	1040	46
13	−	−	+	+	3.5	1135	1193	84
14	+	−	+	+	1.3	1076	1044	89
15	−	+	+	+	2.9	1103	1184	88
16	+	+	+	+	1.2	1040	1027	71

**Table 3:** Chemical analysis of samples based on weight percentage.

Compounds	Samples			
	**A**	**B**	**C**	**D**
Binary Basicity (${\mathrm{CaO/SiO}}_{2}$, wt:wt)	1.03	1.00	1.04	1.01
${\mathrm{Na}}_{2}$O (wt%)	9.40	8.00	8.00	8.00
${\mathrm{TiO}}_{2}$ (wt%)	5.00	6.00	5.40	4.60
F (wt%)	0.00	1.50	3.00	0.00
$B_{2}O_{3}$ (wt%)	0.00	1.90	0.00	0.00
Others (wt%) *	85.60	82.60	83.60	87.40

* Others include ${\mathrm{SiO}}_{2}$ (26–28 wt%), CaO (26–28 wt%), MgO (5–7 wt%), MnO (3–4 wt%), ${\mathrm{Al}}_{2}O_{3}$ (2–4 wt%), C (5 wt%), and impurities.

**Table 4:** Viscosities at 1300 °C and crystallization temperatures.

Characteristics	Samples			
	**A**	**B**	**C**	**D**
Crystallization Temperature (°C)	1301	1069	1159	1309
Viscosity at ${\mathrm{1300}}^{\circ }$C (dPa·s)	2.70	2.97	1.75	4.58

**Table 5:** Main event temperatures observed during heating up.

Sample	T_g_ (°C)	T_cryst_ (°C) on Heating	T_melting range_ (°C)
A	550	670	1000–1270
B	520	730	1030–1270
C	530	700	1010–1300
D	520	720	900–1300

## Data Availability

The original contributions presented in this study are included in the article. Further inquiries can be directed to the corresponding author.

## Abbreviations

The following abbreviations are used in this manuscript:

MDPI	Multidisciplinary Digital Publishing Institute
DSC	Differential Scanning Calorimetry
XRD	X-Ray Diffraction
SEM-EDS	Scanning Electron Microscopy Equipped with Energy Dispersive Spectroscopy
HSLA	High-Strength Low-Alloy
AHSS	Advanced High-Strength Steels
